# Impact of COVID‐19 on Renal Transplant Recipients in a National Transplant Center

**DOI:** 10.1002/iid3.70187

**Published:** 2025-04-18

**Authors:** Pooja Koirala, Pukar Chandra Shrestha, Sugat Ratna Tuladhar, Kalpana Kumari Shrestha, Kopila Dahal

**Affiliations:** ^1^ Department of Nursing Shahid Dharmabhakta National Transplant Centre Bhaktapur Nepal; ^2^ Department of Surgery and Transplantation Shahid Dharmabhakta National Transplant Centre Bhaktapur Nepal; ^3^ Department of Research Shahid Dharmabhakta National Transplant Centre Bhaktapur Nepal; ^4^ Department of Nephrology Shahid Dharmabhakta National Transplant Centre Bhaktapur Nepal

**Keywords:** COVID‐19, infection, mortality, renal transplant, vaccination

## Abstract

**Background:**

Renal transplant recipients are at higher risk of COVID‐19 infection due to chronic comorbidities and immunocompromised state. Limited information is available in Nepal regarding this infection among organ transplantation patients.

**Methods:**

A retrospective cross‐sectional study was conducted on 601 patients to assess the impact of COVID‐19 among patients who underwent renal transplant surgery at Shahid Dharmabhakta National Transplant Center (SDNTC), Bhaktapur. All the renal transplant patients who were COVID‐19 confirmed through PCR tests were included. A self‐developed, semi‐structured telephone interview schedule was utilized for data collection.

**Results:**

Among the patients who responded to our telephone calls, more than a quarter (37.9%) were diagnosed as COVID‐19 positive. The mortality rate was relatively low at 5.7% overall, but significantly higher at 14.9% among those with COVID‐19 positive. Hypertension and diabetes mellitus were the top two comorbid conditions. The common reporting symptom was fever followed by cough. Interestingly, 23.7% of COVID‐19‐positive patients were hospitalized, and among them, 72.2% were admitted for more than 2 days. Notably, 65.8% of the COVID‐19 patients were not vaccinated and among vaccinated ones, only 17.1% of patients were fully vaccinated. The most common vaccine was Vero Cell. There was sixfold increased chance of COVID‐19 infection among unvaccinated patients than vaccinated ones. However, there was no significant association between mortality and age, sex, occupation and vaccination status.

**Conclusion:**

This study highlights the heightened vulnerability of renal transplant recipients to COVID‐19 as significant portion of the studied patients tested positive for COVID‐19, with a notable mortality rate among these patients. The findings underscore the critical role of vaccination, as a considerable number of the COVID‐19 positive patients were unvaccinated.

## 1 Introduction

Renal transplantation is the preferred treatment modality for end‐stage renal disease (ESRD), offering superior survival and quality of life compared to dialysis [1, 2]. However, the success of kidney transplantation relies on lifelong immunosuppression, which increases susceptibility to infections, including viral diseases such as Coronavirus disease 2019 (COVID‐19). Caused by severe acute respiratory syndrome coronavirus 2 (SARS‐CoV‐2), COVID‐19 was declared a Public Health Emergency of International Concern by WHO in January 2020 [3]. As reported by Worldometer, by April 2024, the global tally of confirmed COVID‐19 cases had reached 704 million, with 7.01 million deaths attributed to the virus. In Nepal, during the same period, a total of 1,003,450 infections and 12,031 deaths were recorded [4].

The most severe complication of COVID‐19 typically affects the lungs, with manifestations ranging from asymptomatic or mild pneumonia to severe cases characterized by hypoxia. In critical conditions, patients may develop shock, respiratory failure, multiorgan dysfunction, or even die due to the disease [5]. The COVID‐19 pandemic posed significant challenges for transplant recipients, who, due to their immunocompromised status and a high prevalence of comorbidities like diabetes, heart disease, and lung disease, faced increased risks of severe disease outcomes and elevated mortality rates compared to the general population [6]. In a study looking at COVID‐19 infection in solid organ transplant recipients in Switzerland, the most common presenting symptoms were fever (76%), dry cough (57%), nausea (33%), and diarrhea (33%) while 91% and 24% of patients required hospital and ICU admission, respectively, and 19% were intubated [7]. Another study conducted in Paris, France showed that the mortality rate related to COVID‐19 in renal transplant recipients was 20%–28% as compared to 1%–5% in the general population [8]. A study conducted in India found that most of the renal transplant recipients infected with COVID‐19 required hospitalization and had a poor outcome [9]. During the pandemic, live and deceased donor transplants dropped by 39.5% and 11.9%, respectively, while the overall transplant rate decreased by 19.1% [10]. Although several such studies have explored the impact of COVID‐19 in transplant recipients, most have been conducted in high‐income countries with well‐established healthcare infrastructures. Data on COVID‐19 infections in renal transplant recipients from low‐ and middle‐income countries (LMICs), remain scarce. Understanding the burden, risk factors, and outcomes of COVID‐19 among kidney transplant recipients is essential for clinical management and preventive strategies for this vulnerable group in the future.

Nepal, with its unique demographic and healthcare constraints, presented a distinct setting where COVID‐19 may have disproportionately affected transplant recipients [11]. The limited healthcare accessibility, variability in post‐transplant care, and delays in COVID‐19 vaccination among transplant patients in Nepal further underscore the need for a focused investigation [12]. Nepal's vaccination program began in January 2021, with COVISHIELD for frontline and essential workers, followed by Vero Cell from April 2021 for the general population through public health centers. Later, Johnson & Johnson targeted vulnerable groups, and Pfizer was provided to children aged 12–17 [13]. Nepal's vaccination program primarily relied on donations and COVAX supplies and faced a vaccine shortage in mid‐2021 [13]. On the other hand, in developed nations vaccination programs began in December 2020, with Pfizer‐BioNTech, Moderna and AstraZeneca vaccine with faster rollouts, broader access, and consistent vaccine availability [14, 15].

Considering these scenarios, this study aims to assess the prevalence, risk factors, vaccination status, and clinical outcomes of COVID‐19 among renal transplant recipients from Shahid Dharmabhakta National Transplant Center (SDNTC), Nepal. To the best of our knowledge, no prior study has examined the prevalence of COVID‐19 in renal transplant patients in Nepal, nor has any research explored their vaccination status. The only related study [16] to date focused on the mortality of renal transplant recipients with COVID‐19; however, its study population was confined to patients under the care of the specific hospital where the research was conducted. In contrast, our current study adopts a broader approach, covering all patients who had undergone transplantation at SDNTC, regardless of whether they continued their follow‐up care at our center during or post‐COVID‐19 infection. Consequently, our study includes participants from all provinces of Nepal. To our knowledge, this may be the first study to document the vaccination status—including vaccine types, doses, and coverage—of renal transplant patients in this population.

The primary objective of the study was to determine the prevalence of COVID‐19 among renal transplant patients, clinical presentations, assess their vaccination status, hospitalization and evaluate the outcomes of COVID‐19 in infected individuals. The secondary objective was to explore potential associations between various socio‐demographic factors, vaccination status, and COVID‐19 infection outcomes in this population.

## 2 Methods

This quantitative descriptive retrospective study considered all patients who had undergone renal transplant at Shahid Dharmabhakta National Transplant Center (SDNTC) from 2013 January to 2021 January (*n* = 800). Both living and deceased donor renal transplant patients were included, while renal transplant recipients with diagnosed graft failure or patients on dialysis were excluded from the study.

COVID‐19 infection was defined as the patients with positive PCR test result. To assess the impact of COVID‐19 in our renal transplant patients, the researchers developed a semi‐structured questionnaire in consultation with peers and experts. The questionnaire consisted of two parts:

- Part I comprised of questions related to the socio‐demographic information (age, sex, address and occupation), years of transplant, existing co‐morbidities, COVID‐19 infection and vaccination status.
- Part II addressed COVID‐19‐specific aspects: clinical features, duration of hospitalization, and outcome.

Responders for which testing was performed before instrument validation (*N* = 80) were excluded from the study. Adjustments to the questionnaire were made based on pretest findings. Validity and reliability of the instrument were tested through peer and expert reviews. The Shapiro–Wilk test was used to assess data normality, yielding a P‐value of 0.002, indicating a non‐normal distribution.

Ethical approval for the study was obtained from the Ethical Review Board of the Nepal Health Research Council, Nepal (approval no. 65/2022). Additionally, formal permission was obtained from the Shahid Dharmabhakta National Transplant Center (SDNTC), Bhaktapur. Patient data, including names, dates of birth, transplant dates, and phone numbers, were retrieved from the medical records department at SDNTC. Surveys were conducted via phone calls between September and October 2022, using the developed questionnaire. All calls were made from the hospital's official number, and the calls were recorded for documentation purposes.

First, the identity of the patient was verified by asking for the date of birth and year of transplantation. Then, verbal informed consent was obtained. After consent was secured, the survey questions were administered, with each call lasting approximately 10–15 min. In case the patients had expired, we asked the questions to a first degree relative after taking the informed consent from them.

Given the retrospective nature of the study, there might have been chances of recall bias from the patient side when answering the questions asked about COVID‐19 infection by the researchers. To minimize it, respondents were given ample time to recall and respond to the questions.

Confidentiality of the data was strictly maintained by ensuring that only the researchers had access to the information and by reassuring respondents that the data collected would be used exclusively for research purposes. Following data collection, the information was reviewed for accuracy and completeness before being entered into Excel 2018 and subsequently exported to the Statistical Package for Social Sciences (SPSS) version 16 for analysis.

Data were analyzed by using descriptive (mean, percentage, and frequency) and inferential statistics (*χ* ^2^ test and logistic regression). The study was conducted as per the STROBE guidelines.

## 3 Results

**Figure 1:**
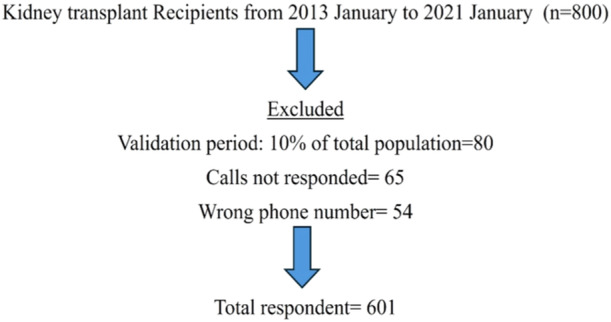
Cohort selection flowchart.

Out of the total 800 kidney transplant recipients, 601 were included in this study (Figure 1). Among these 601 renal transplant recipients, 228 (37.9%) had contracted COVID‐19.

The characteristics of this cohort of patients, at the time of survey, based on presence or absence of COVID‐19 disease are listed in Table 1. Of all COVID‐19‐positive patients in our cohort, majority (79.8%) were male, with a similar sex ratio in the COVID‐19‐negative group. COVID‐19 cases were relatively evenly distributed across the different age groups, with no single age group significantly over‐represented. The largest number of patients in both groups resided in Province 3. Nearly half (48.2%) of the COVID‐19‐positive patients were within 3 years of transplant. The most common (38.6%) comorbid condition among the COVID‐19‐positive patients was hypertension. A higher proportion of COVID‐19‐positive patients were unvaccinated (65.8%), while most COVID‐19‐negative patients were vaccinated (76.4%). *χ* ^2^ test showed a significant association between vaccination status and infection rates (*p* < 0.001).

**Table 1:** Baseline characteristics of the renal transplant recipients according to the presence of COVID‐19 disease.

Characteristics	All patients,	COVID‐19 positive patients,	COVID‐19 negative patients,	*p* value
	*n* = 601	*n* = 228 (37.9%)	*n* = 373 (62.0%)	
Sex				
Male	480 (79.9%)	182 (79.8%)	298 (79.9%)	0.984
Female	121 (20.1%)	46 (20.2%)	75 (20.1%)	
Age group in years				
15–25	90 (15.0%)	46 (20.2%)	44 (11.8%)	
25–35	166 (27.6%)	56 (24.6%)	110 (29.5%)	0.036
35–45	180 (30.0%)	63 (27.6%)	117 (31.4%)	
> 45	165 (27.5%)	63 (27.6%)	102 (27.3%)	
Address				
Province 1	107 (17.8%)	57 (25.0%)	50 (13.4%)	
Province 2	94 (15.6%)	49 (21.5%)	45 (12.1%)	
Province 3	189 (31.4%)	71 (31.1%)	118 (31.6%)	< 0.001
Province 4	92 (15.3%)	23 (10.1%)	69 (18.5%)	
Province 5,6,7	119 (19.8%)	28 (12.3%)	91 (24.4%)	
Occupation				
Working	371 (61.7%)	143 (62.7%)	228 (61.1%)	0.679
Not working	230 (38.3%)	85 (37.3%)	145 (38.9%)	
Years of transplantation				
1–3 years	200 (33.3%)	110 (48.2%)	90 (24.1%)	
3–6 years	264 (43.9%)	88 (38.6%)	176 (47.2%)	< 0.001
> 6 years	137 (22.8%)	30 (13.2%)	107 (28.7%)	
Comorbid conditions				
Diabetes	56 (9.3%)	20 (8.8%)	36 (9.7%)	
Hypertension	216 (35.9%)	88 (38.6%)	128 (34.3%)	
Diabetes and Hypertension	75 (12.5%)	40 (17.5%)	35 (9.4%)	< 0.001
Others	18 (3%)	13 (5.7%)	5 (1.4%)	
None	236 (39.6%)	67 (29.4%)	169 (45.4%)	
Vaccination status				
Not vaccinated	238 (39.6%)	150 (65.8%)	88 (23.6%)	< 0.001
Vaccinated	363 (60.4%)	78 (34.2%)	285 (76.4%)	

Among the vaccinated recipients, Vero cell was the most common (74%) vaccine and around two‐thirds were fully vaccinated (Table 2).

**Table 2:** Vaccine name and coverage among the vaccinated recipient (*n* = 363).

	Variable	Frequency (%)
Vaccine name		
Vero cell	First dose	125 (34.4)
	Second dose (full dose)	144 (39.6)
Johnson & Johnson	First does (full dose)	86 (24.0)
Pfizer	First dose	8 (2.0)
Vaccine coverage	Partially vaccinated	133 (36.4)
	Fully vaccinated	230 (63.6)

Among the 228 COVID‐19‐positive renal transplant recipients, 85.08% recovered, while 14.9% expired (Table 3). Patient aged 35–45 years demonstrated high recovery (32.0%) and low mortality (2.9%). Patients within 3 years of transplant showed the highest recovery rate (54.1%), whereas those within 3–6 years of post‐transplantation experienced the highest mortality (76.5%). The most common presenting symptom was fever, which was reported in 117 of 228 (51.3%) patients. Among the COVID‐19‐positive patients, who recovered one‐third (36.1%) of them were asymptomatic, and the majority (85.6%) did not require hospitalization. There was a significant association between the outcome of COVID‐19 and age, address, years of transplant, clinical presentation, and hospitalization (Table 3).

**Table 3:** Baseline characteristics of the COVID‐19 positive renal transplant recipients according to the outcome of disease.

Characteristics	All patients,	Recovered,	Expired,	*p* value
	*n* = 228	*n* = 194 (85.08%)	*n* = 34 (14.9%)	
Age group in years				
15–25	46 (20.2%)	35 (18.0%)	11 (32.4%)	
25–35	56 (24.6%)	48 (24.7%)	8 (23.5%)	
35–45	63 (27.6%)	62 (32.0%)	1 (2.9%)	0.002
> 45	63 (27.6%)	49 (25.3%)	14 (41.2%)	
Sex				
Male	182 (79.8%)	153 (78.9%)	29 (85.3%)	0.389
Female	46 (20.2%)	41 (21.1%)	5 (14.7%)	
Address				
Province 1	57 (25.0%)	51 (26.3%)	6 (17.6%)	
Province 2	49 (21.5%)	42 (21.6%)	7 (20.6%)	
Province 3	71 (31.1%)	69 (35.6%)	2 (5.9%)	< 0.001
Province 4	23 (10.1%)	18 (9.3%)	5 (14.7%)	
Province 5, 6, 7	28 (12.3%)	14 (7.2%)	14 (41.2%)	
Occupation				
Working	143 (62.7%)	121 (62.4%)	22 (64.7%)	0.795
Not working	85 (37.3%)	73 (37.6%)	12 (35.3%)	
Years of transplantation				
1–3	110 (48.2%)	105 (54.1%)	5 (14.7%)	
3–6	88 (38.6%)	62 (32.0%)	26 (76.5%)	< 0.001
> 6	30 (13.2%)	27 (13.9%)	3 (8.8%)	
Clinical presentation				
Cough	26 (11.4%)	19 (9.8%)	7 (20.6%)	
Fever	117 (51.3%)	94 (48.5%)	23 (67.6%)	< 0.001
Others	15 (6.6%)	11 (5.7%)	4 (11.8%)	
Asymptomatic	70 (30.7%)	70 (36.1%)	0 (0.0%)	
Hospitalization				
Yes	54 (23.7%)	28 (14.4%)	26 (76.5%)	< 0.001
No	174 (84.5%)	166 (85.6%)	8 (23.5%)	
Duration of hospitalization (*n* = 54)				
1–2 days	15 (27.8%)	10 (35.7)	5 (19.2%)	0.177
> 2 days	39 (72.2%)	18 (64.3%)	21 (80.8%)	
Vaccination status				
Not vaccinated	150 (65.8)	132 (68.0%)	18 (52.9%)	0.08
Vaccinated	78 (34.2%)	62 (31.9%)	16 (47.1%)	

The factors associated with COVID‐19 infection included being aged 15–25 years (OR: 1.69, *p* = 0.04), residing in Province 1 (OR: 3.71, *p* < 0.001), Province 2 (OR: 3.54, *p* < 0.001), or Province 3 (OR: 1.96, *p* = 0.01). Additionally, individuals with hypertension (OR: 1.73, *p* = 0.006), both diabetes and hypertension (OR: 2.88, *p* < 0.001), or other underlying diseases (OR: 6.56, *p* = 0.001) were at higher risk. Those within 3 years post‐transplantation (OR: 2.44, *p* < 0.001) and unvaccinated individuals (OR: 6.22, *p* < 0.001) also had a greater likelihood of infection (Table 4).

**Table 4:** Factors associated with COVID‐19 infection in renal transplant recipients. (*N* = 601).

			Multivariate analysis	
Factors associated with COVID‐19 infection	Number of patients	Number of COVID‐19 positive patients	Odds ratio (95% CI)	*p* value
Age group in years				
15–25	90	46	1.69 (1.01–2.84)	0.04
25–35	166	56	0.82 (0.53–1.29)	0.40
35–45	180	63	0.87 (0.56–1.34	0.54
> 45	165	63	1	
Sex				
Male	480	182	1.03 (0.655 to1.621)	0.90
Female	121	46	1	
Address				
Province 1	107	57	3.71 (2.1–6.54)	< 0.001
Province 2	94	49	3.54 (1.97–6.36)	< 0.001
Province 3	189	71	1.96 (1.17–3.28)	0.01
Province 4	92	23	1.08 (0.57–2.04)	0.80
Province 5,6,7	119	28	1	
Occupation				
Working	371	143	1.07 (0.76–1.5)	0.70
Not working	230	85	1	
Comorbid conditions				
Diabetes mellitus	56	20	1.4 (0.76–2.59)	0.28
Hypertension	216	88	1.73 (1.17–2.57)	0.006
Diabetes and hypertension	75	40	2.88 (1.69–4.92)	< 0.001
Others	18	13	6.56 (2.25–19.11)	0.001
None	236	67	1	
Years of transplantation				
1–3	200	110	2.44 (1.67–3.57)	< 0.001
3–6	264	88	0.56 (0.35–0.91)	0.02
> 6	137	30	1	
Vaccination status				
Not vaccinated	238	150	6.22 (4.331–8.957)	< 0.001
Vaccinated	363	78	1	

Regarding COVID‐19 infection outcomes, in comparison to patients residing in Province 3, patients from all other provinces had a significantly higher risk of mortality. Additional factors linked to increased mortality included more than 6 years post‐transplantation (OR: 4.26, *p* < 0.004) and requiring hospitalization due to COVID‐19 infection (OR: 19.27, *p* < 0.001) (Table 5).

**Table 5:** Factors associated with outcome COVID‐19 infections in renal transplant recipients (*n* = 228).

			Multivariate analysis	
Factors associated with outcome COVID‐19 infections	Number of COVID‐19 Positive patients	Number of expired patients due to COVID‐19	Odds ratio (95% CI)	*p* value
Age group in years				
15–25	46	11	1.187 (0.377–3.741)	0.77
25–35	56	8	0.444 (0.132–1.495)	0.19
35–45	63	1	0.050 (0.005–0.478)	0.01
> 45	63	14	1	
Sex				
Male	182	29	1.49 (0.57–3.94)	0.42
Female	46	5	1	
Address				
Province 1	57	6	5.55 (1.1–28.03)	0.04
Province 2	49	7	7.52 (1.53–36.96)	0.01
Province 3	71	2	1	
Province 4	23	5	5.37 (1.02–28.25)	0.047
Province 5,6,7	28	14	12.47 (2.78–55.92)	< 0.001
Occupation				
Working	143	22	1.15 (0.56–2.36)	0.71
Not working	85	12	1	
Years of transplantation				
1–3	110	5	1	
3–6	88	26	0.87 (0.21–3.72)	0.85
> 6	30	3	4.26 (1.61–11.3)	0.004
Hospitalization				
Yes	54	26	19.27 (7.93–46.82)	< 0.001
No	174	8	1	
Vaccination status				
Not vaccinated	150	18	0.53 (0.25–1.11)	0.09
Vaccinated	78	16	1	

## 4 Discussion

This study provides new insight into COVID‐19 infection among renal transplant recipients, in one of the developing countries where annually around 300 renal transplants are performed. In this study, out of the 601 transplant recipients included, 228 (37.9%) had contracted COVID‐19. The overall mortality rate in the studied population was 5.7%, whereas among those who tested positive for COVID‐19 14.9% expired due to complications related to the infection. A study done among the general population in Nepal showed that the total COVID‐19 positive cases until May 2022 was 979,076 (3.3%), and mortality was 0.04%, which is far lower than the rate observed in our study [11]. The higher infection rate in our cohort may be attributed to the immunocompromised state and the presence of various comorbid diseases in transplanted patients. Another study from 2022, conducted among renal transplant recipients at a Teaching Hospital in Kathmandu, Nepal, reported that 11.8% of these patients tested positive for COVID‐19, with a mortality rate of 21% among those infected [16]. The difference in COVID‐19 positive rate observed in this study may be due to the small sample size of 71 as compared to the sample size of 601 taken in the current study. Also, the study [16] was undertaken in one of the biggest tertiary care center of Nepal, so more sick and seriously ill patients may be presented there compared to our center.

Around 62% of the COVID‐19‐positive patients in our study were employed. The higher infection rate in this group may be due to the challenges in maintaining social distancing at the workplace and the necessity of frequently leaving home for work‐related activities.

In our cohort, the most common vaccine was Vero cell, followed by Johnson and Johnson and Pfizer. COVISHIELD was the first vaccine rolled out in Nepal; however, it was given to frontline and essential workers. This could be the reason that none of the transplant recipient in our study received this vaccine.

One of the important findings of this study is the association between vaccination status and COVID‐19 infection. Our analysis found that unvaccinated renal transplant recipients were six times more likely to be infected with COVID‐19 compared to their vaccinated counterparts. This finding reinforces the protective role of immunization in this vulnerable population. Given that Nepal's vaccination program faced supply challenges and delays [13], these findings underscore the need for improved vaccine accessibility and targeted immunization efforts for immunocompromised individuals.

In terms of outcomes following infection, among patients who died from COVID‐19, 52.9% were unvaccinated. This aligns with findings from studies conducted in India [12], Italy [17], USA [18], and Nepal [19], which suggest that vaccination plays a role in reducing mortality and the severity of COVID‐19 infection. However, other factors such as age, general health, social distancing measures, comorbidities, and the use of immunosuppressive medications may also contribute to variations in mortality and outcomes.

Another important observation we made was that patients within the first 3 years post‐transplantation had a significantly higher risk of infection. This finding aligns with other studies which have shown that transplant recipients in their early postoperative period (< 2 years) were prone to COVID‐19 infection [20], indicating that higher doses of immunosuppressants in early post‐transplant years could contribute to increased susceptibility to infections [6].

In our study, the presence of comorbid conditions, particularly hypertension and diabetes, and other conditions (such as hypothyroidism and asthma), was strongly associated with COVID‐19 infection risk. Similar data was reported from a 23 hospital multicenter study from across India, which showed that the most common comorbid condition among COVID‐19 positive renal transplant recipients was hypertension (95%) followed by diabetes (55%) [21]. These data reinforce the importance of monitoring and managing these conditions in transplant recipients. An important finding related to comorbid conditions was that more than a quarter (29.4%) of the renal transplant recipient did not have any disease at the time of COVID‐19 infection. This proportion represents those patients who had some disease that necessitated a transplant; however, the disease was treated along with transplantation. For example: In some patients the disease leading them to needing transplantation was kidney stone. In these patients, kidney stone was removed together with transplantation. So, at the time of the survey they did not have any other disease.

Our study also found that fever was the most common presenting symptom (51.3%), consistent with other studies. This finding can be compared with a similar study conducted in Nepal and India, which showed that the common symptom was fever (86%) and cough (84%) [9, 16, 21]. Studies conducted in France and USA also showed that the most common presenting symptom was fever followed by cough [6, 8]. Interestingly, the rate of asymptomatic cases (30.7%) in our study was higher than reported in other studies, suggesting possible underreporting or milder presentations in our setting.

Regarding the hospitalization of patients due to COVID‐19 complications, in our study, around a quarter, that is, 23.7% of them were hospitalized. Among the hospitalized ones, 72.2% of them were hospitalized for more than 2 days. On the contrary, a study conducted in India and USA found that 52.2% and 78% of post‐renal transplant patient were hospitalized [6, 9]. Another study conducted in Nepal showed that 25.5% of them were admitted for less than 7 days [16]. The difference in the findings may be due to the difference in the data collection technique and instrumentation. As in all the above published study data was collected among patients visiting OPD and ER while in the current study, data was collected among all the renal transplant recipients of a transplant center via phone call interview.

Geographic disparities were also evident in our study, with patients from Provinces 1, 2, and 3 demonstrating significantly higher odds of COVID‐19 infection. These findings reflect regional differences in healthcare infrastructure, population density, and public health interventions. Similarly, mortality rates varied by province, with the highest mortality observed in Provinces 5, 6, and 7, where healthcare resources are more limited. These disparities highlight the need for region‐specific health policies and resource allocation to ensure equitable access to care.

Our study has some limitations like (1) The study included adult patients, most of which are recipients of a living donor kidney transplant, therefore our findings might be difficult to generalize for other age groups and recipients of deceased donor kidney transplants. (2) Information was collected via telephone interviews, so there might be chances of recall bias. (3) Patients were asked about their vaccination status at a single period of time so relationship between pre‐/post‐vaccination and COVID‐19 infection as well as mortality may be different.

## 5 Conclusion

Based on the study findings, it is concluded that more than a quarter of renal transplant recipients were diagnosed as COVID‐19 positive, and the overall mortality rate was higher than the general population in Nepal. Patients with short duration of transplantation have a higher chance of contracting COVID‐19 infections. Only about a quarter of COVID‐19‐infected patients were vaccinated and among them, very few them were fully vaccinated. The most common vaccine used was Vero cell. There was a decreased chance of contracting COVID‐19 among vaccinated patients than unvaccinated ones. Mortality was not significantly associated with age, sex, and vaccination status. The finding of the study might be useful to the SDNTC and Ministry of Health and Population (MOHP) in providing baseline data for their record and in case of emergence of similar pandemic in the future, to integrate information about risk factors in renal transplant recipients to help balance benefits and risks and better advise patients about potential risks.

## Author Contributions

**Pooja Koirala:** conceptualization, formal analysis, methodology, writing – original draft, writing – review and editing. **Pukar Chandra Shrestha:** conceptualization, data curation, investigation, supervision. **Kalpana Kumari Shrestha:** project administration, resources, software. **Kopila Dahal:** resources, software, visualization, writing – review and editing.

## Conflicts of Interest

The authors declare no conflicts of interest.

## Data Availability

The data used to support the findings of the study are available from the corresponding author upon request and approval of the ethical committee.

## References

[1] A. D. Barlow and A. S. Ghoneima , “Kidney Transplantation,” Surgery (Oxford) 41, no. 9 (2023): 596–602, 10.1016/j.mpsur.2023.06.005.

[2] F. Sidra Farishta , S. Hassan Sajjad , Z. Ahmad Zeb , K. Akash Kumar , N. Zara Nisar , and A. Amer Azhar , “KIDNEY TRANSPLANT: Reasons for Preference of Kidney Transplantation Versus Hemodialysis by End Stage Kidney Disease Patients,” Journal of Saidu Medical College, Swat 12, no. 4 (2022): 169–173, 10.52206/jsmc.2022.12.4.741.

[3] S. Chauhan , “Comprehensive Review of Coronavirus Disease 2019 (Covid‐19),” Biomedical Journal 43, no. 4 (2020): 334–340, 10.1016/j.bj.2020.05.023. 32788071 PMC7263230

[4] “COVID‐19 Coronavirus Pandemic,” COVID‐Coronavirus Statistics—Worldometer, accessed November 28, 2023, https://www.worldometers.info/coronavirus/.

[5] M. Gavriatopoulou , E. Korompoki , D. Fotiou , et al., “Organ‐Specific Manifestations of COVID‐19 Infection,” Clinical and Experimental Medicine 20, no. 4 (2020): 493–506, 10.1007/s10238-020-00648-x. 32720223 PMC7383117

[6] V. Nair , N. Jandovitz , J. S. Hirsch , et al., “COVID‐19 in Kidney Transplant Recipients,” American Journal of Transplantation 20, no. 7 (2020): 1819–1825, 10.1111/ajt.15967. 32351040 PMC7267603

[7] J. Tschopp , A. G. L'Huillier , M. Mombelli , et al., “First Experience of SARS‐CoV‐2 Infections in Solid Organ Transplant Recipients in the Swiss Transplant Cohort Study,” American Journal of Transplantation 20, no. 10 (2020): 2876–2882, 10.1111/ajt.16062. 32412159 PMC7272999

[8] M. Elias , D. Pievani , C. Randoux , et al., “COVID‐19 Infection in Kidney Transplant Recipients: Disease Incidence and Clinical Outcomes,” Journal of the American Society of Nephrology 31, no. 10 (2020): 2413–2423, 10.1681/ASN.2020050639. 32847984 PMC7609004

[9] P. Jha , S. Shukla , D. Bansal , et al., “COVID‐19 in Renal Transplant Recipients—A Single Center Experience From India,” Indian Journal of Nephrology 32, no. 5 (2022): 416, 10.4103/ijn.IJN_479_20. 36568590 PMC9775611

[10] O. Aubert , D. Yoo , D. Zielinski , et al., “COVID‐19 Pandemic and Worldwide Organ Transplantation: A Population‐Based Study,” Lancet Public Health 6, no. 10 (2021): e709–e719, 10.1016/S2468-2667(21)00200-0. 34474014 PMC8460176

[11] S. K. Adhikari , K. Ranabhat , S. Bhattarai , et al., “Epidemiology of COVID‐19 Mortality in Nepal: An Analysis of the National Health Emergency Operation Center Data,” Public Health Challenges 2, no. 4 (2023): e127, 10.1002/puh2.127. PMC12039602 40496798

[12] B. Ahluwalia , N. K. Gupta , A. Singh , et al., “Effect of COVID‐19 Vaccination Status on Outcome of Adult Patients Admitted at a Tertiary Care Centre in India,” Monaldi Archives for Chest Disease 93, no. 1 (2022), 10.4081/monaldi.2022.2135. 35443571

[13] S. Chhetri , S. Manandar , S. Chhetri , et al., “Enablers and Barriers of COVID‐19 Vaccination Efforts in Nepal,” Public Health Challenges 2, no. 1 (2023): e67, 10.1002/puh2.67. PMC12039702 40496964

[14] S. E. Oliver , M. Wallace , E. Twentyman , et al., “Development of COVID‐19 Vaccine Policy—United States, 2020–2023,” Vaccine 42 (2024): 125512, 10.1016/j.vaccine.2023.12.022. 38158297 PMC11893158

[15] R. Link‐Gelles , A. Britton , and K. E. Fleming‐Dutra , “Building the U.S. COVID‐19 Vaccine Effectiveness Program: Past Successes and Future Directions,” Vaccine 42 (2024): 125492, 10.1016/j.vaccine.2023.12.002. 38129285 PMC11304400

[16] N. Bhurtyal and D. S. Shah , “COVID‐19 Infection in Renal Transplant Recipients: Experience From a Tertiary Care Center in Nepal,” Journal of Institute of Medicine Nepal 44, no. 1 (2022): 60–67, 10.59779/jiomnepal.1209.

[17] G. Russo , Q. Lai , L. Poli , et al., “SARS‐COV‐2 Vaccination With BNT162B2 in Renal Transplant Patients: Risk Factors for Impaired Response and Immunological Implications,” Clinical Transplantation 36, no. 1 (2022): e14495, 10.1111/ctr.14495. 34569101 PMC8646240

[18] V. Atanasov , N. Barreto , J. Whittle , et al., “Understanding COVID‐19 Vaccine Effectiveness Against Death Using a Novel Measure: COVID Excess Mortality Percentage,” Vaccines 11, no. 2 (2023): 379, 10.3390/vaccines11020379. 36851256 PMC9959409

[19] A. Karmacharya , K. Rai , S. Siwakoti , B. Khanal , and N. R. Bhattarai , “COVID‐19 Breakthrough Infections in Vaccinated Individuals at Bpkihs, Nepal,” BMC Infectious Diseases 24, no. 1 (2024): 1003, 10.1186/s12879-024-09902-z. 39300352 PMC11411789

[20] E. Kolla , A. Weill , M. Zaidan , et al., “COVID‐19 Hospitalization in Solid Organ Transplant Recipients on Immunosuppressive Therapy,” JAMA Network Open 6, no. 11 (2023): e2342006, 10.1001/jamanetworkopen.2023.42006. 37934496 PMC10630896

[21] G. Bhandari , V. Tiwari , A. Gupta , et al., “COVID‐19 Infection in Renal Transplant Patients: Early Report From India,” Indian Journal of Nephrology 31, no. 3 (2021): 271, 10.4103/ijn.IJN_323_20. 34376942 PMC8330666

